# Healtheatre: Drama and Medicine in Concert

**DOI:** 10.3390/healthcare5030037

**Published:** 2017-07-28

**Authors:** Ian K. Walsh, Paul Murphy

**Affiliations:** 1 Centre for Medical Education, Queen’s University Belfast School of Medicine, Belfast BT9 7BL, UK; 2 Drama Department, School of Arts, English and Languages, Queen’s University Belfast, Belfast, BT7 1NN, UK; p.murphy@qub.ac.uk

**Keywords:** drama and healthcare, interdisciplinary learning, simulation, Stanislavski system

## Abstract

**Introduction:** Clinical practice includes expressing empathy and understanding key features of humanity, such as mortality and illness. The Stanislavski “System” of actor training negotiates a journey from the unconscious via feeling, will and intellect to a proposed supertask. This study explored these areas during collaborative learning amongst undergraduate medical and drama students. **Materials and Methods:** Each of two interactive sessions involved teams of final year medical students rotating through challenging simulated clinical scenarios, enacted by undergraduate drama students, deploying key techniques from the Stanslavski system of actor training. Team assessment of performance was via a ratified global scoring system and dynamic debriefing techniques. **Results:** Medical students reported an enhanced immersive experience within simulated clinical scenarios. Drama students reported increased challenge and immersion within their roles. Medical faculty and standardised patients reported positive utility and value for the approach. Clinical team assessment scores increased by 47% (*p* < 0.05) with this intervention. **Discussion:** Qualitative and quantitative data demonstrated the merit and utility of such interdisciplinary learning. All students and faculty appreciated the value of the activity and described enhanced learning. Collaborative dynamic debriefing allowed for a continuation of the immersive experience and allowed for an exploration of arenas such as empathy. **Conclusions:** The deployment of drama students trained in the Stanislavski system significantly enriched medical and drama student experience and performance. Team assessment scores further demonstrated the effectiveness of this approach. Feedback from students, faculty and standardised patients was uniformly positive. The approach facilitated exploration of empathy.

## 1. Introduction

Clinical practice includes demonstrating empathy and understanding key features of humanity, such as illness and mortality. Recent high-profile reports addressing patient safety issues have highlighted a disturbing lack of compassion amongst doctors [1]. An apparent emphasis on emotional detachment and a focus on maintaining clinical neutrality, together with overly deployed attendant technology may limit human interaction in early medical undergraduate education [2]. There have been attempts to address these shortfalls by inculcating actor training and dramaturgical techniques to medical education, with variable degrees of reported success [3,4].

The Stanislavski “System” of actor training allows creative union between actor and character, which thus has the potential to explore and develop empathy. The approach negotiates a journey from the unconscious via feeling, will and intellect to a proposed Supertask (the character’s purpose within the play’s context), as part of a comprehensive “psycho-physical” approach to representing the complexity of the human experience on stage [5]. The term “psycho-physical” emphasises the holistic approach advocated by Stanislavski in terms of placing equal weight on both the psychological and physical aspects when developing a role in rehearsal and then performing that role in a play with other actors. Creativity lies at the heart of the System, insofar as the actor is encouraged to draw upon their own life experience in developing the character, as well as imagining what it would be like if they were the character in terms of their emotional life, in order to create an authentic performance that will elicit an empathic response from the audience. The approach begins with a conscientious reading of the play text, which in this study involved a standardized clinical scenario that was adapted into a short vignette from which the drama students could develop their respective characters. The drama students analysed the facts and figures in the text in order to develop the ‘Given Circumstances’ of the play in which they could locate their character by taking into consideration: the story; the conditions of life for the character; the temporal setting; the physical setting; the actors’ and director’s interpretation of the play; the technical details of the production including set, costumes, props, lighting and sound. The information gleaned from the Given Circumstances enables the actor to develop subtle idiosyncrasies of voice and movement known as the ‘Subtext’ that gives the character a unique quality, which in turn enhances the sense of realism.

The character is further augmented with the use of the “Magic If”, which encourages the actor to use their imagination to consider what they would think, feel and do “if” they were the character in a particular situation. In one of clinical scenarios for example, the drama students were required to act as if they were a patient who has been prepared for the operating room to have a planned sigmoidoscopy. The patient has been waiting for this procedure for several months and is very anxious, because both the patient’s father and younger brother died from bowel cancer. The surgeon who was scheduled to perform the sigmoidoscopy has asked the doctor (performed by the medical student) to talk to the patient to advise that the procedure has been cancelled due to a major incident requiring the use of several operating rooms, and that another appointment will be sent out to re-attend for the procedure. The drama student, therefore, has to imagine how they would feel “if” they were the patient in terms of the fear, frustration, indignation, anger and associated emotional states they would experience if they were in that situation. The Magic If draws the actor out of their own situation and into that of the character in the scenario, and encourages them to act in terms of how they would respond to the information they received about their cancelled operation. The Magic If facilitates an imaginative response to the possibilities of the Given Circumstances without demanding a prescribed and therefore artificial response.

The actor adds even greater depth to their character by using a tool known as Emotion Memory, variously known also as “affective memory” and “emotion recall”. Emotion Memory works through the imagination by linking past-tense memories with future-tense projections. Emotion Memory is a veritable treasure-trove for the actor, insofar as real-life experiences can be drawn upon to enhance the fictional scenario surrounding the character. Emotion Memory transforms the actor from the objective position of looking at the character’s experience to the subjective position of enacting the character’s experience; for example, in terms of waiting for an operation, learning that they have a life-limiting illness, witnessing the death of a family member and all the other variegated experiences that one encounters throughout life.

The deployment of tools such as Given Circumstances, Magic If and Emotion Memory enables the actor to organise the relevant information into a logical sequence of “Bits” of action that comprise individual “Tasks” which in turn culminate in the realisation of the “Supertask” or purpose of the character within the overall scope of the play. The emphasis on creativity is key to the process of building and performing a role, especially as clinical scenarios and the short vignettes are not play texts with lines that can be memorised, but are scenarios that require drama students to improvise specifically in terms of an action-reaction-decision sequence. The maxim “all acting is reacting” is useful in explaining the mechanics of the interactive engagement between the drama students and medical students as they perform their respective roles in terms of the complexities of the action, reaction, decision process which drive the respective scenario.

From a drama or performance perspective, these dramaturgical issues were explored in this study by means of collaborative, interdisciplinary learning amongst undergraduate medical and drama students.

## 2. Methods 

The Patient Safety module (MED5018) within the final year of study at Queen’s University School of Medicine encompasses teaching and learning relating to technical and nontechnical clinical skills, with an emphasis on human factors, including ergonomics. Toward the end of each iteration of the module, students participate in clinical teams within simulated clinical scenarios that are specifically scripted and designed to address challenging scenarios within the human factors arena. Formative teaching of nontechnical clinical skills within these scenarios is reinforced by summative scoring of clinical team performance, derived from global scores provided by tutors/facilitators, extant simulated patients and team peers. Scores are generated from standardized marking templates, quantifying both technical and nontechnical skill demonstration (nontechnical, interactive skills include communication, teamworking, leadership, active followership, situational awareness and decision making). Attention to these areas occurs initially during the debriefing period within each simulated scenario. During the wider discussion arena (“Grand Rounds”) after all simulations have been completed, further discussion and exploration is directed towards key issues of higher clinical functioning, such as cognition, metacognition and empathy.

Preparation: Prior to engaging in the clinical scenarios with the medical students, the drama students watched two OSCE training videos. The videos respectively displayed a “good” OSCE and a “bad”, together with the roles of medical student and doctor facilitating the OSCE, as performed by medical professionals, with a service-user performing the role of a simulated patient. After observing a standardized OSCE analysis, the drama students were facilitated by drama and clinical academic staff to develop a means by which they could bring their understanding of acting and performance to analyzing the roles and training videos.

For the study itself, each of two interactive sessions involved 20 final year medical students, with teams of 4 rotating through challenging simulated clinical scenarios, enacted by 17 rotating undergraduate drama students, deploying the Stanslavski System of actor training.

The drama students then provided detailed feedback to the medical students during the debriefing session following each scenario when they took part in the collaborative engagement in the Patient Safety module (MED5018). Additionally, the drama students were able to offer detailed feedback during the Grand Rounds debriefing session involving all groups at the training session. 

Assessment of clinical team performance within simulated scenarios was via a ratified global scoring system and dynamic debriefing techniques, as part of the standard operation procedure during the Patient Safety module outwith the current study.

Qualitative data was initially generated when all participating students (both drama and medical) were asked to comment on the quality and content of the intervention. To enhance relevance and validity, triangulation of data was gleaned by incorporating quality outcome and student satisfaction questionnaire surveys, which are part of the standard evaluation for the Patient Safety module (MED5018); these are provided after each iteration of the module (the module runs in 12 weekly tranches). In addition to free text comments, responses were ranked on a standardized 5-point Likert scale to provide metric evaluation. Similar triangulation was generated by incorporating the module evaluations for the drama module Theatre and Social Intervention (DRA3057).

## 3. Results

### 3.1. Student Feedback

Feedback comments on the intervention were consistently encouraging from both medical and drama students alike. Whilst negative comment was invited, none was received. Individual comments were received from 12 drama and 10 medical students. Examples of specifically positive comments are highlighted in the below quotations:

“I found the interschool learning with the drama students invaluable. It added another dimension to the scenarios with some fantastic acting.”

—PT, medical student

“It was a fantastic experience to bring our art into real life situations which in turn assisted the medical students”

—CH, drama student

“The drama students were great. They made scenarios realistic and gave us the opportunity to interact in a professional capacity with people of our own age group. It would be great to have more of this throughout the 5 years.”

—FW, medical student

“I think as drama students we can learn a lot about improvisation and naturalistic acting by working alongside the medics in their OSCE assessments. Thanks so much for the opportunity! Very beneficial.”

—SD, drama student

The benefits of interdisciplinary learning were thus evident from both student groups, from a variety of perspectives; ranging from the advantage of working with age-relatable peers to enhanced realism within simulated scenarios.

### 3.2. Module Evaluation Questionnaires

Within the module evaluation questionnaires (which included opportunity to suggest areas for improvement), there were similarly encouraging responses from both student groups. Questionnaire responses were received from 80% of students overall. Examples of specific positive comments are again highlighted below:

“It was helpful to deal with people the same age as ourselves, and who offered a fresh take on the roles they were playing. Including more of the same in future events would be a real bonus.”

—Medical student A

“The interaction between drama students, bringing our skills of acting into another course at Queen’s. The practical side worked well from the drama students’ point of view. I think this could be used in other education based modules.”

—Drama student A

“This is probably the best week of teaching that I've had during my 5 years at Queen's. The simulation training is so beneficial and would be of great use throughout the UG course. I felt the sessions with the drama student were great! Their acting skills and also being fresh to medical jargon made them more likely to call us up on using it.”

—Medical student B

“Working with other schools at Queen’s has been fantastic and extremely interesting. Everything with regards assessment has always been made clear.”

—Drama Student B

**Figure 1:**
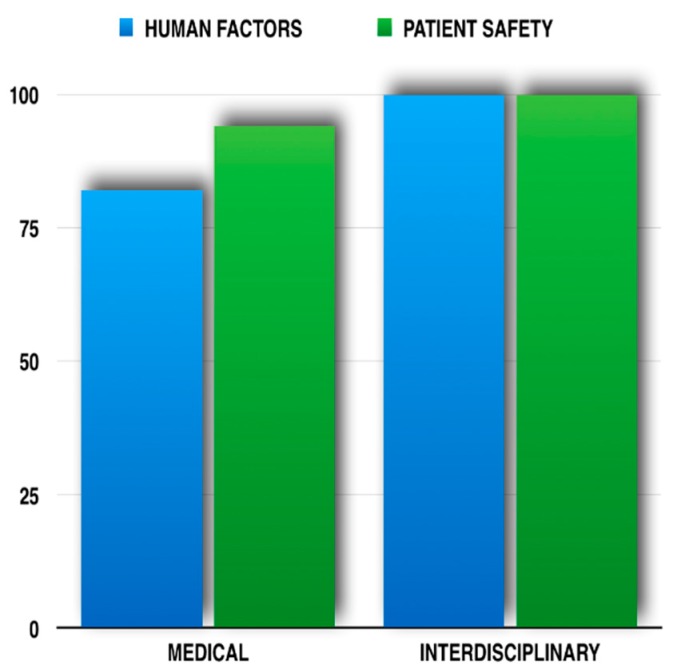
Module Feedback (80% response rate)—Student perception of improved knowledge (5-point Lickert scale) in: (**Left**) Human Factors specifically and (**Right**) Patient Safety in general.

Medical student perceptions of improved knowledge relating specifically to human factors and patient safety in general are demonstrated in Figure 1. Most students thus agreed or strongly agreed that the addition of drama students to the educational mix enhanced learning in both these domains.

### 3.3. Clinical Team Performance

**Figure 2:**
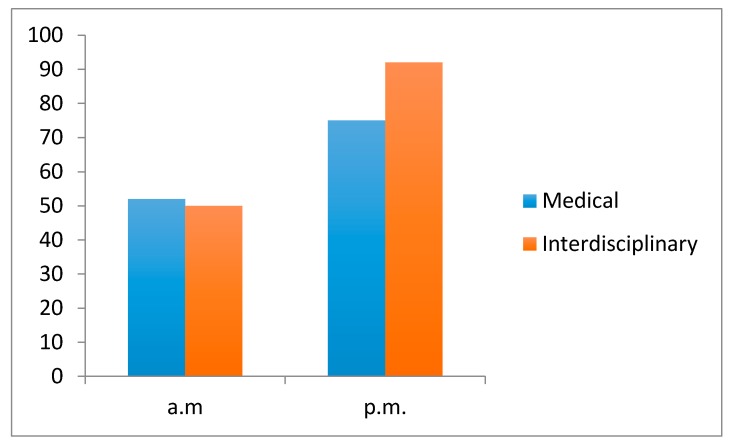
Clinical Team Performance. Performance scores increased by 47% (*p* < 0.05) with the addition of drama students.

Assessment of clinical team performance scores are evidenced in Figure 2. For all student teams, there was a statistically significant (Student’s *t*-test; *p* < 0.05) improvement in performance scores from a baseline of mean score of 52% in the morning simulation scenarios, compared with a mean score of 75% for the afternoon sessions. With the interdisciplinary intervention, there was a further statistically significant improvement to a mean performance score of 92%. This equated to a 47% improvement in clinical performance rating when drama students were added to the clinical scenarios. 

### 3.4. Debriefing: Empathy

Standardised, facilitator-led debriefing took place as an integral component of each enacted clinical scenario, immediately upon scenario completion. Standardised patients and drama students as simulated patients were both encouraged to provide performance assessment scores and contribute generically to the debrief, by providing comments on team and individual clinical performance and interaction.

The “Grand Rounds” sessions took place immediately following a refreshment break, after all simulated scenarios had been completed. Drama students gave feedback as a form of advocacy for their patient by acting partly “in role”, whereby they spoke with the patient’s voice and displayed behaviours consistent with that patient. This altered the dynamics of the standard debrief, which usually involves standardised patients (out of role) passing brief comment on team and individual performance. These debrief dynamics allowed for deeper discussion of issues such as empathy and professionalism. Maintaining “in role” status allowed for greater involvement of the “patient” during debrief, facilitating discussions of areas such as nonverbal communication and healthcare practitioner’s attitudinal approach.

## 4. Discussion

This educational intervention was aimed at improving simulation-based education (SBE) in patient safety within the medical undergraduate curriculum. The approach was innovative from three particular perspectives:

(1) Whilst experiential theatre training has been effectively deployed in this arena, the deployment of Stanislavskian methodology within SBE has been reported sparingly [3,4].

(2) The interdisciplinary approach was new to the medical undergraduate curriculum at Queen’s. To date, the closest approximation to this at this institution has been the application of interprofessional education within the healthcare undergraduate sector (typically as medical, nursing and/or pharmacy students learning together in simulated clinical scenarios). Such interprofessional or multiprofessional healthcare education is commonplace nowadays [6]. The approach described here is distinct, insofar as it is truly *interdisciplinary* learning for student groups within what have been traditionally considered as somewhat disparate student groups.

(3) The profoundly immersive experience from medical students’ perspective, together with the advocacy role demonstrated by drama students when “in role” allowed for a uniquely dynamic exploration of key issues relating to the human condition within healthcare, such as empathy. Enhanced empathic communication has been reported with the utilisation of actor training for medical undergraduates [7]. However, our study was distinctive in examining the enhancement of empathy via such simulated patient advocacy.

The qualitative and quantitative data outlined in the preceding sections demonstrated the merit and utility of such interdisciplinary learning. Both groups of students and faculty appreciated the value of the activity and described enhanced learning. Whilst student comment and feedback was persistently positive, further exploration of such encouraging responses with expanded questionnaire facilities or focus groups may have provided more meaningful and realistic data, as well as suggestions for improvement.

Medical students consistently reported an enhanced immersive experience when the drama students were introduced to the simulated scenarios. Such deep levels of immersion during simulated clinical scenarios have been shown to significantly enhance simulation-based learning experience and improve educational effectiveness [8,9].

Collaborative dynamic debriefing allowed for a continuation of the immersive experience and facilitated an exploration of arenas such as empathy. Beyond nontechnical skill acquisition, typified by communication and teamworking, it would appear that this approach has the potential to gauge and foster such higher levels of clinical functioning within medical undergraduates. This is an often overlooked area in curricular development and one considered integral to the concept of professionalism [10,11].

Quantification of the effects of the intervention was provided by team assessment scores, generated from standardised scoring templates, providing metrics on technical skills (e.g., knowledge, efficiency, fluency) and nontechnical skills (e.g., communication, teamworking, situational awareness). This data further demonstrated the effectiveness of the interdisciplinary approach.

The deployment of drama students trained in the Stanislavski system thus significantly enriched medical and drama student experience and performance. Feedback from students, faculty and standardised patients was uniformly positive. The approach facilitated explorations of empathy. The effectiveness of the collaboration between the medical and drama students in terms of the interactive clinical scenarios lay in the uniqueness of each performance, insofar as the tools deployed from the Stanislavskian system enabled the drama students to improvise by reacting creatively to the actions of the medical students, thus achieving a greater sense of realism. It would seem that this sense of uniqueness borne of creative improvisation is the dimension which sets the interactive scenarios outlined in this study from the more scripted, standardized approach; whereby simulated patients perform in a more predictable and therefore less realistic manner. We hope that further investigation of this interdisciplinary arena may prove beneficial in preparing senior medical undergraduates for effective clinical practice in the current era of patient safety.

## 5. Conclusions

1. Interdisciplinary learning involving medical and drama students is effective and provides for rich experiential learning.
2. Clinical team performance is enhanced by this approach.
3. Deployment within dynamic debriefing allows for exploration of key issues such as empathy.
